# An adaptive fusion of composite attention convolutional neural network for polyp image segmentation

**DOI:** 10.3389/fphys.2025.1678403

**Published:** 2026-01-07

**Authors:** Bojiao Jin, Yi Zhang, Qianqing Nie, Lin Qi, Wei Qian

**Affiliations:** Northeastern University, Shenyang, China

**Keywords:** adaptive feature fusion, convolutional attention, depth-wise separable convolution, gating units, polyp segmentation

## Abstract

**Background:**

Accurate localization and segmentation of polyp lesions in colonoscopic images are crucial for the early diagnosis of colorectal cancer and treatment planning. However, endoscopic imaging is often affected by noise interference. This includes issues like uneven illumination, mucosal reflections, and motion artifacts. To mitigate the impact of such interference on segmentation performance, it is essential to integrate multi-scale feature analysis effectively. Features at different scales capture distinct aspects of image information. Yet, existing methods typically rely on simple feature summation or concatenation. These methods lack the capability for adaptive fusion across scales.

**Methods:**

To address these limitations, this paper proposes AFCNet—an Adaptive Fusion Composite Attention Convolutional Neural Network. AFCNet is designed to improve robustness against noise interference and enhance multi-scale feature fusion for polyp segmentation. The key innovations of AFCNet include: (1) integrating depthwise separable convolution with attention mechanisms in a multi-branch architecture. This allows for the simultaneous extraction of fine details and salient features. (2) Constructing a dynamic multi-scale feature pyramid with learnable weight allocation for adaptive cross-scale fusion.

**Results:**

Extensive experiments on five public datasets (ClinicDB, Kvasir-SEG, etc.) demonstrate that AFCNet achieves state-of-the-art performance, with improvements of up to 3.73 % in Dice coefficient and 4.62 % in IoU, validating its effectiveness and generalization capability in polyp segmentation tasks.

**Conclusion:**

AFCNet is a novel polyp segmentation network that leverages convolutional attention and adaptive multi-scale feature fusion, delivering exceptional generalization and adaptability.

## 1 Introduction

Colorectal cancer is a common malignant tumor with an increasing incidence rate, posing a serious threat to human health. Therefore, the prevention of colorectal cancer has become an important focus of medical research. Studies have shown that polyps are often precancerous lesions in colorectal cancer. Early detection and removal of colorectal polyps is one of the most effective methods for reducing the incidence of colorectal cancer and improving cure rates (Jia et al., 2019). Physicians rely on screening tools such as colonoscopy for the diagnosis of colon cancer. However, in clinical practice, small polyps may be missed by the naked eye, potentially delaying timely treatment (Zimmermann-Fraedrich et al., 2019). Automatic and precise polyp segmentation can assist doctors in precisely locating polyp regions within the colon (Guo et al., 2020), enhancing diagnostic accuracy and reducing the likelihood of oversight. Therefore, polyp segmentation plays a crucial role in the early diagnosis of colorectal cancer.

Due to the complex shapes and varying sizes of polyps, effectively fusing multi-scale features is crucial for significantly enhancing the model’s segmentation performance. Deep learning-based techniques have driven advancements in colon polyp segmentation. Convolutional neural network (CNN)-based approaches, such as U-Net (Ronneberger et al., 2015) and its variants, including UNet++ (Zhou et al., 2019) and Unet3+ (Huang et al., 2020), improve performance through nested skip connections. However, these methods are inadequately modeling long-range dependencies and rely on relatively simple integration strategies for fusing features from different scales. As a result, they may introduce noise from low-level information, and high-level features can blur the boundary details preserved in low-level features.

Transformer-based approaches (e.g., Polyp-pvt (Dong et al., 2021), MSRAformer (Wu et al., 2022), and SSFormer (Wang et al., 2022)) demonstrate superior feature extraction capabilities, but still face two challenges: (a) insufficient attention to the importance of features during the decoding process, and (b) suboptimal integration of information across different scales. Recently, researchers have proposed hybrid methods that combine CNNs and Transformers to leverage the strengths of both (Peng et al., 2024). However, existing approaches have not fully considered the potential multi-scale features within the same layer and the issue of semantic mismatch between features that are far apart in the hierarchy.

This paper proposes a U-shaped polyp segmentation network architecture based on convolutional attention and multi-scale feature adaptive fusion. Extensive experiments demonstrate that our method outperforms existing polyp segmentation approaches in both segmentation accuracy and generalizability across five colorectal polyp datasets. The paper makes two key contributions: (1) A new Multi-scale Depth-wise Convolutional Attention Module (MDCA): the MDCA module consists of a depth-separable convolutional and multi-branching network, which extracts multi-scale features within the layer and enhances the focus and utilization of important features. (2) A new Multi-scale Adaptive Feature Fusion Module (MAFF), which consists of a multi-scale cross-fusion network and an Adaptive Multi-Scale Feature Harmonization (AMFH) module. The multi-scale cross-fusion network enables smooth transmission of feature information across semantic hierarchies through a progressive feature fusion approach. Additionally, the adaptive multi-scale feature coordination module provides a flexible way to integrate and strengthen feature information at different levels.

The rest of the paper is organized as follows. Section 2 systematically reviews the related research work in the field of polyp segmentation and analyses the advantages and shortcomings of the existing methods. Section 3 comprehensively describes the network architecture design of AFCNet, and thoroughly analyses the implementation principles and technological breakthroughs of the three core modules, namely, MDCA, MAFF and UFR. Section 4 describes the experimental setup in detail, including dataset configuration, evaluation indexes and comparative experimental design, and analyses the results quantitatively and qualitatively. Finally, Section 5 gives the conclusions of this paper.

## 2 Related work

### 2.1 Polyp segmentation network

Traditional segmentation algorithms such as Otsu’s method (Vala and Baxi, 2013), Region Growing (Pohle and Toennies, 2001), Snake (Bresson et al., 2007) and other methods are sensitive to noise and image quality. Additionally, setting and adjusting their parameters is difficult, and they often provide insufficient segmentation accuracy and fail to capture fine details. Consequently, these methods yield low segmentation accuracy for polyps. In contrast, deep learning methods can automatically learn complex image features, handle noise more robustly, and eliminate the need for manual parameter tuning (Ahamed et al., 2024b).

Thus, deep learning methods provide more accurate and robust segmentation results in many application scenarios Ahamed et al. (2023a). With the development of Convolutional Neural Networks (CNN), especially with the introduction of U-Net (Ronneberger et al., 2015), many models inspired by this architecture have shown promising results in the field of medical image segmentation. UNet reduces the resolution of an image through a series of convolutional and pooling layers to capture the contextual information of the image. It then gradually restores the resolution using upsampling and convolution operations, effectively combining low- and high-resolution features to enable precise pixel-level segmentation. EU-Net Patel et al. (2021) enhances semantic information by introducing a global context module for extracting key features. ACSNet (Zhang et al., 2020) modifies the skip connections in U-Net into a local context extraction module and adds a global information extraction module. CENet (Gu et al., 2019) uses a ResNet pre-trained model as an encoder for feature extraction, fused with a context extraction module. It relies on Dense Cavity Convolutional Block (DAC module) and Residual Multi-Kernel Pooling (RMP module) to capture more abstract features and preserve spatial information, leading to improved medical image segmentation performance.

Although CNN has been successful in the field of polyp segmentation, it has limitations in acquiring contextual remote information. Transformer, as a powerful image-understanding method, makes up for this deficiency well and is rapidly developing in the field of polyp segmentation. Polyp-pvt (Dong et al., 2021) the first to introduce the Transformer as a feature encoder for polyp segmentation. It integrates high-level semantic and positional information through cascading fusion modules and similarity aggregation modules, effectively suppressing noise in the feature representations. DuAT (Tang et al., 2023), a dual-fusion Transformer network, employs a global-to-local spatial aggregation module to combine global and local spatial features, thereby enabling precise localization of polyps of varying sizes. In addition, it employs a selective boundary aggregation module to fuse the edge information at the bottom layer with the semantic information at the top layer. SSFormer (Wang et al., 2022) combines Segformer (Xie et al., 2021) and Pyramid Vision Transformer as an encoder and introduces a new progressive local decoder to emphasize the local features and alleviate the problem of distraction. TransNetR (Jha et al., 2024) combines the residual network with the Transformer. The combination shows good real-time processing speed and multi-center generalization capability.

### 2.2 Attention mechanism

By precisely focusing on key regions of an image, the attention mechanism enables deep learning models to identify polyps more efficiently and accurately, particularly in colonoscopy images. Att-UNet (Lian et al., 2018) integrates Attention into UNet and applies it to medical images, and for the first time, incorporates Soft Attention into a CNN network for medical imaging. DCRNet (Yin et al., 2022) proposes a positional attention module to capture pixel-level contextual information. PraNet (Fan et al., 2020) aggregates high-level features using a parallel partial decoder, exploits boundary cues using a reverse attention module, and establishes relationships between regions and boundary. MultiResUNet (Ahamed et al., 2024a) extracts features at different scales through multi-resolution convolutional blocks, and uses attention guidance to enhance focus on polyp regions, significantly improving the segmentation performance of colorectal polyps. CaraNet (Lou et al., 2022) combines axial reverse attention and channel feature pyramid (CFP) modules to improve the segmentation performance of small medical targets. MSRF-NET (Srivastava et al., 2021) uses a dual-scale dense fusion block to exchange multi-scale features with different receptive fields. It maintains the resolution and propagates high-level and low-level features for more accurate segmentation outcomes.

ResNest (Zhang et al., 2022) is an innovative architecture that combines the Residual Network (ResNet) with a split-attention mechanism, and has demonstrated excellent performance in semantic segmentation. By introducing the split-attention module—which effectively integrates grouped convolution with attention mechanisms—ResNeSt enables the network to more effectively capture and utilize both spatial and channel-wise features, while maintaining computational efficiency. However, its application in the field of polyp segmentation has not been explored in depth. In this paper, ResNeSt is employed as an advanced CNN backbone to assess its potential in polyp segmentation tasks and to evaluate the effectiveness and generalizability of the proposed modules.

### 2.3 Feature fusion

Due to the complex shapes and varying sizes of polyps, effectively fusing multi-scale features can significantly enhance the model’s segmentation performance. DCRNet (Yin et al., 2022) achieves feature enhancement by embedding a contextual relationship matrix and then achieves relationship fusion by region cross-batch memory. MSNet (Zhao et al., 2021) introduces a phase reduction unit to extract differential features between adjacent layers and employs a pyramid structure with varying receptive fields to capture multi-scale information. CFA-Net (Zhou et al., 2023) uses a hierarchical strategy to incorporate edge features into a two-stream segmentation network while using a cross-layer feature fusion module to fuse neighboring features across different levels. Work such as PPNet (Hu et al., 2023) and PolypSeg (Zhong et al., 2020) apply attention mechanisms to enhance feature fusion between the top and bottom layers. Gating mechanisms have also proven effective for feature fusion, as demonstrated by Gated Fully Fusion (Li et al., 2020) and BANet (Lu et al., 2022), which selectively integrate multi-level features through gated fusion. Collectively, these works demonstrate that efficiently fusing and utilizing extracted features is a promising method in polyp segmentation.

## 3 Methods

**FIGURE 1:**
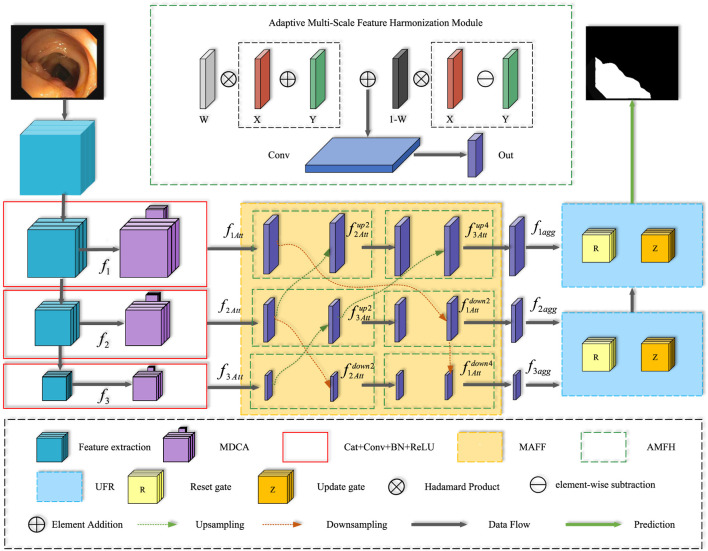
The AFCNet network framework consists of four key parts. The processing pipeline flows from the Encoder Network, to the MDCA, then to the MAFF, and finally to the UFR.

In this section, we provide a detailed overview of the architecture of the AFCNet network and its constituent modules. Firstly, the overall structure of the network is presented in Figure 1.

We then describe each component in detail, including the Multi-Scale Depth-wise Convolution Attention Module (MDCA module), the Multi-Scale Adaptive Feature Fusion module (MAFF), and the Upsampling Feature Retrospective Module (UFR).

### 3.1 Network architecture

The AFCNet we designed follows the classical encoder-decoder architecture. For the encoding part of the model, we employ the traditional CNN network Res2Net50 as the backbone. We use the first three layers of high-level features extracted from the backbone network. Suppose our input polyp segmentation image is $F\in R^{H\times W}$ . We utilize the feature information of each level $f_{k}\in R^{\frac{H}{2^{k+1}}\times \frac{W}{2^{k+1}}}\left(k\in \left[1,3\right]\right)$ . The Multi-scale Depth-wise Convolutional Attention Module (MDCA) applies convolutional attention mechanisms to feature information at different hierarchical levels, gathering key information within the image while suppressing less significant elements. The MDCA module enhances the model’s feature representation for each pixel point in the input image by capturing multi-scale information through convolutional kernels of different sizes. Moreover, the enhanced attentional features and the original features are effectively fused in this module by a dense concatenation operation.

After subsequent enhancement of features by MDCA, the features $f_{k}^{m}$ are input into the Multi-scale Adaptive Feature Fusion (MAFF) module. Within the MAFF module, a cross-network aligns features of different scales. Subsequently, the Adaptive Multi-Scale Feature Harmonization (AMFH) module performs weighted fusion on the adjusted feature maps, emphasizing differences and key information within the features to heighten the model’s sensitivity to image details. Through a 3 × 3 convolution, features across various scales are efficiently integrated. Finally, the multi-scale fused feature information is processed through a specially designed UFR, effectively integrating features from different network layers while considering their dynamic interrelations, leading to the final segmentation prediction map. Our overall network structure is defined in Equations 1–4:

$$f_{k}=Res2Net\left(F\right),k\in \left[1,3\right]$$ (1)

$$f_{k_{\mathrm{Att}}}=MDCA\left(f_{k}\right),k\in \left[1,3\right]$$ (2)

$$f_{k_{\mathrm{agg}}}=MAFF\left(f_{k_{\mathrm{Att}}}^{m},W_{i}\right),k\in \left[1,3\right],i\in \left[1,6\right]$$ (3)

$$F_{\mathrm{out}}=UFR\left(f_{k_{\mathrm{agg}}}\right)$$ (4)

### 3.2 Multi-scale depth-wise convolution attention module

In order to extract more important feature information from different layers, the MDCA module is designed in this paper. This module consists of a multi-branch parallel network and a multi-scale deep convolutional attention mechanism. This module first integrates feature information from multiple receptive fields within each layer, ensuring that the output of each layer simultaneously captures detailed, local contextual, and global semantic information. By introducing an internal multi-scale feature extraction and fusion module prior to inter-level feature fusion, the representation quality and richness of single-layer features are greatly enhanced. This design establishes a progressive fusion paradigm—first optimizing the internal structure and then coordinating external relationships—allowing the network to achieve smoother and more controllable feature evolution from local details to global semantics. Ultimately, this improves both the accuracy of complex boundary segmentation and the model’s generalization ability.

**FIGURE 2:**
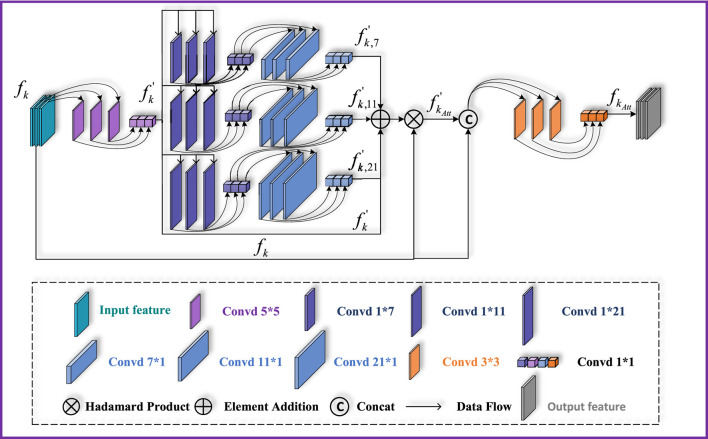
Structure of the MDCA module. It consists mainly of depth-wise separable convolution and a multi-branch depth-wise dilated convolution structure.

As shown in Figure 2, the features $f_{k}$ are obtained from the encoder. First, $f_{k}$ is convolved by a depth-separable convolution with a convolution kernel size of 5×5 to obtain the spatial feature $f_{k}^{\prime }$ . The obtained features $f_{k}^{\prime }$ are then fed into a multi-branch concurrent network structure consisting of three different branches. And there are two depth directions of banded depth-separable convolution in each branch. The size of the depth-separable convolution kernel in each branch is set to 7, 11, and 21, respectively. Capturing multi-scale contextual information in each branch through these different orientations and sizes of convolutions enables the network to capture a wider range of contextual information in the image and to better understand the image features at different spatial scales. Thus, this design enhances the network’s sensitivity to objects with diverse shapes and structures. We define depth-separable convolution in Equation 5:

$$DWSConv_{m\times n}\left(f\right)=\phi \left(Conv_{m\times n}\left(f\right)\right)$$ (5)

Where ϕ(⋅) stands for point-by-point convolution, and $Conv_{m\times n}$ stands for convolutional layers with convolutional kernel size m×n . After the multi-branch network fully extracts image information, attention maps $f_{k,7}^{\prime },f_{k,11}^{\prime },f_{k,21}^{\prime }$ are obtained from different branches. The attention feature maps are then summed from different branches and multiplied with the input feature maps for feature optimization to obtain $f_{K_{\mathrm{Att}}}^{\prime }$ . Finally, the module uses splicing to fuse the optimized features with the original features in the channel through an information aggregation stage, followed by a final $3^{*}$ 3 convolution. The module integrates rich multi-scale information to enhance the model’s representation of contextual features. Mathematically, the MDCA module can be described by the Equations 6–11:

$$f_{k}^{\prime }=DWSConv_{5\times 5}\left(f_{k}\right)$$ (6)

$$f_{k,7}^{\prime }=DWSConv_{7\times 1}\left(DWSConv_{1\times 7}\left(f_{k}^{\prime }\right)\right)$$ (7)

$$f_{k,11}^{\prime }=DWSConv_{11\times 1}\left(DWSConv_{1\times 11}\left(f_{k}^{\prime }\right)\right)$$ (8)

$$f_{k,21}^{\prime }=DWSConv_{21\times 1}\left(DWSConv_{1\times 21}\left(f_{k}^{\prime }\right)\right)$$ (9)

$$f_{k_{\mathrm{Att}}}^{\prime }=f_{k}⊗\left(f_{k}^{\prime }+f_{k,7}^{\prime }+f_{k,11}^{\prime }+f_{k,21}^{\prime }\right)$$ (10)

$$f_{k_{\mathrm{Att}}}=\psi \left(BN\left(L_{3\times 3}\left(\left(f_{k_{\mathrm{Att}}}^{\prime },f_{k}\right)\right)\right)\right)$$ (11)

where $f_{k}\left(k\in \left[1,3\right]\right)$ is a different hierarchical characterization of the input, $DWSConv_{m\times n}$ is depth-wise convolution, Concat represents the feature concatenation operation. ψ(⋅) means ReLU function, BN denotes batch normalization, $L_{3\times 3}\left(\cdot \right)$ means $DWSConv_{3\times 3}$ and Concat.

### 3.3 Multi-scale Adaptive Feature Fusion Module

Due to the low contrast between polyps and surrounding tissues in some polyp endoscopic images, features extracted by traditional methods may have difficulty in distinguishing subtle differences between polyps and normal tissues. To fully leverage features at different scales and enhance the richness of feature representation, we propose a Multi-scale Adaptive Feature Fusion (MAFF) module. This method introduces a progressive, hierarchical feature fusion approach. As illustrated in Figure 1, this model establishes a series of intermediate representations between feature layers with significant semantic gaps, using them to guide the information flow with finer granularity between layers. This ensures a smooth transition from spatial details to semantic concepts, helping to alleviate the feature mismatch problem between different semantic levels.

MAFF consists of two main components: a multi-scale fusion cross-network and an Adaptive Multi-scale Feature Harmonization module. The multi-scale fusion cross-network realizes dynamic interaction and complementarity between different scale features through its unique structure, providing a basis for the model to capture rich, multi-level information. At the core of MAFF is the Adaptive Multi-Scale Feature Harmonization module, which comprises two distinct operations: a feature addition unit and a feature subtraction unit. Feature addition is a commonly used feature enhancement algorithm in the image domain, and in our module, the common information present in different levels of features is highlighted by performing addition operations on the features at different levels. The opposite feature subtraction unit is able to highlight the differences in information between features at different levels. In order to fully fuse these two complementary feature information, we introduce a trainable weighting ratio parameter, $W_{i}$ . With the trainable parameter $W_{i}$ , the module is able to achieve fine control of the feature fusion process, thus enhancing the model’s generalization ability and robustness to different endoscopic images.

The MAFF module receives inputs $f_{k_{\mathrm{Att}}}$ (k∈[1,3]) , which are multi-scale enriched features output from the MDCA module. These features are first processed by the Multi-Scale Fusion Cross-Network, where bilinear interpolation is used to align the spatial scales through upsampling and downsampling. Convolutional layers are then applied to further refine the feature representations.

This process can be mathematically expressed in Equations 12–14:

$$UP_{k}\left(f\right)=\psi \left(BN\left(Conv_{3\times 3}\left(B_{k}\left(f\right)\right)\right)\right.$$ (12)

$$Down_{k}\left(f\right)=\psi \left(BN\left(Conv_{3\times 3}\left(B_{\frac{1}{k}}\left(f\right)\right)\right)\right.$$ (13)

$$\left\{\begin{array}{cc} f_{1_{\mathrm{Att}}}^{\mathrm{down2}} & =Down_{2}\left(f_{1_{\mathrm{Att}}}\right)\\ f_{1_{\mathrm{Att}}}^{\mathrm{down4}} & =Down_{4}\left(f_{1_{\mathrm{Att}}}\right)\\ f_{2_{\mathrm{Att}}}^{\mathrm{up2}}= & UP_{2}\left(f_{2_{\mathrm{Att}}}\right)\\ f_{2_{\mathrm{Att}}}^{\mathrm{down2}} & =Down_{2}\left(f_{2_{\mathrm{Att}}}\right)\\ f_{3_{\mathrm{Att}}}^{\mathrm{up2}}= & UP_{2}\left(f_{3_{\mathrm{Att}}}\right)\\ f_{3_{\mathrm{Att}}}^{\mathrm{up4}}= & UP_{4}\left(f_{3_{\mathrm{Att}}}\right) \end{array}\right.$$ (14)

where $Conv_{3\times 3}$ means the operation that consists of a sequence of 3 × 3 convolution, BN means batch normalization, and ψ is the ReLU function. B denotes the sampling method of bilinear interpolation.

We then put the aligned features into the AMFH (Adaptive Multi-scale Feature Harmonization) module. AMFH fuses two different features by feature addition and subtraction in order to efficiently capture the complementary information between different layers of features, highlight the subtle differences between them, and strengthen the module’s sensitivity to edges, textures, and other key visual details. We then enable the module to dynamically balance the effects of addition and subtraction operations on the final feature representation by introducing an adaptive weighting mechanism. This adaptivity is based on the unique properties of the input features and their contextual information, and the optimization of the weights is performed automatically. With the adaptive adjustment of the weights of addition and subtraction operations, the AMFH module takes full advantage of the complementary strengths of these two operations to produce feature representations that are rich and fine-grained. We use X and Y as input features to the AMFH module, defining the AMFH function in Equation 15:

$$AMFH\left(X,Y\right)=\psi \left(BN\left(Conv_{3\times 3}\left(\left|W_{i}⊗\left(X⊕Y\right)+\left(1-W_{i}\right)⊗\left(X⊖Y\right)\right|\right)\right)\right)$$ (15)

where ⊕ is the element-by-element addition operation, ⊖ is the element-by-element subtraction operation, ⊗ is the Hadamard product, $W_{i}$ is the trainable parameter we set i∈[1,6] , |⋅| computes the absolute value, where $Conv_{3\times 3}$ means the operation that consists of a sequence of 3 × 3 convolution, BN means batch normalization and ψ is ReLU function. After the AMFH module we can get three final outputs in Equation 16:

$$\left\{\begin{array}{ccc} f_{1_{\mathrm{agg}}}= & \;AMFH\left(AMFH\left(f_{1_{\mathrm{Att}}},f_{2_{\mathrm{Att}}}^{\mathrm{up2}}\right),f_{3_{\mathrm{Att}}}^{\mathrm{up4}}\right)\\ f_{2_{\mathrm{agg}}}= & \;AMFH\left(AMFH\left(f_{1_{\mathrm{Att}}}^{\mathrm{down2}},f_{2_{\mathrm{Att}}}\right),f_{3_{\mathrm{Att}}}^{\mathrm{up2}}\right)\\ f_{3_{\mathrm{agg}}}= & \;AMFH\left(AMFH\left(f_{1_{\mathrm{Att}}}^{\mathrm{down4}},f_{2_{\mathrm{Att}}}^{\mathrm{down2}}\right),f_{3_{\mathrm{Att}}}\right) & \end{array}\right.$$ (16)

### 3.4 Upsampling Feature Retrospective Module

**FIGURE 3:**
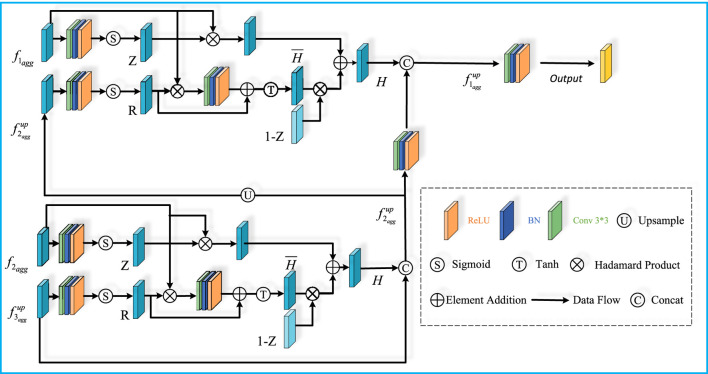
Upsampling Feature Retrospective Module structure. It consisits Update gate unit, reset gate unit and dense connections,the module uses a bilinear interpolation method to upsample features.

After obtaining the fused features, in order to dynamically adjust the amount of information fused in each scale so as to realize more effective information integration, reduce spatial distortion, and enhance the semantic expression of the features in multi-scale feature fusion. We have designed the Up-sampling Feature Retrospective Module (UFR) based on the idea of the Gate Recurrent Unit (GRU). As shown in Figure 3.

In the gated loop unit, the gating mechanism is used to control the flow of information through the sequence model. We input different levels of features into the UFR module, respectively. The UFR module consists of an update gate module and a reset gate module, as well as a dense connection, which performs correlation enhancement of the different levels of features through update gates and reset gates. We set the two inputs of the module to be two neighboring features of different levels: X and Y. Then the update gates and the reset gates are computed by the following Equations 17–20:

$$Z=\sigma \left(\psi \left(BN\left(Conv_{3\times 3}\left(X\right)\right)\right)\right)$$ (17)

$$R=\sigma \left(\psi \left(BN\left(Conv_{3\times 3}\left(Up_{2}\left(Y\right)\right)\right)\right)\right)$$ (18)

$$\bar{H}=T\left(R+\psi \left(BN\left(Conv_{3\times 3}\left(R⊗X\right)\right)\right)\right)$$ (19)

$$H=Z⊗X+\left(1-Z\right)⊗\bar{H}$$ (20)

where σ(⋅) denotes Sigmoid function, ψ(⋅) denotes ReLU function, T(⋅) denotes Tanh function. The obtained hidden vector H is used as one of the outputs of this layer and the inputs of the next layer.

In our module, we up-sample the bottom layer features by using linear interpolation so as to align with the dimensions of the top layer features. We define the above computational process as the G(⋅) function. Our upsampling part can be expressed by Equations 21–24:

$$f_{3_{\mathrm{agg}}}^{up}=UP_{2}\left(f_{3_{\mathrm{agg}}}\right)$$ (21)

$$f_{2_{\mathrm{agg}}}^{up}=C_{3\times 3}\left(G\left(f_{2_{\mathrm{agg}}},f_{3_{\mathrm{agg}}}^{up}\right)\right)$$ (22)

$$f_{1_{\mathrm{agg}}}^{up}=C_{3\times 3}\left(G\left(f_{1_{\mathrm{agg}}},f_{2_{\mathrm{agg}}}^{up}\right)\right)$$ (23)

$$output=\psi \left(BN\left(Conv_{3\times 3}\left(f_{1_{\mathrm{agg}}}^{up}\right)\right)\right)$$ (24)

where $C_{3\times 3}$ denotes Convolution with 3×3 convolution kernel and Concat.

## 4 Experiment, result and discussion

In this section, we provide detailed descriptions of our experiments, including the datasets used and the experimental results. This includes comparisons with 11 widely used methods as benchmarks, along with ablation studies and generalization experiments to validate the effectiveness of our approach.

### 4.1 Experiment

#### 4.1.1 Dataset

According to the (Mei et al., 2023), we selected five publicly available datasets commonly used in the field of polyp segmentation: Kvasir-SEG, CVC-ClinicDB, CVC-ColonDB, CVC-300, and ETIS.

Kvasir-SEG (Jha et al., 2020): It is an open-access dataset of gastrointestinal polyp images and the corresponding segmentation masks, manually annotated and verified by an experienced gastroenterologist. It contains 1,000 polyp images and their corresponding ground truth from the Kvasir-SEG Dataset v2. The resolution of the images contained in Kvasir-SEG varies from 332 × 487 to 1920 × 1,072 pixels.

CVC-ClinicDB (Bernal et al., 2015): CVC-ClinicDB is a database of frames extracted from colonoscopy videos. These frames contain several examples of polyps. The CVC-ClinicDB dataset contains 612 images cut from 25 colonoscopy videos with an image size of 384 × 288 and polyps ranging from 0.34 % to 45.88 % in size.

CVC-ColonDB (Tajbakhsh et al., 2015): The CVC-ColonDB dataset consists of 380 images cut from 15 colonoscopy videos with an image size of 574 × 500 and the polyp size of 0.30 % –63.15 % .

ETIS (Silva et al., 2014): ETIS contains 196 images cut from 34 colonoscopy videos with the image size of 1,225 × 996. The highest resolution compared to other datasets. But the size of polyps in its images is only 0.11 % –29.05 % , the smallest, making this dataset also more challenging.

CVC-300 (Vázquez et al., 2017): includes 60 colonoscopy images with a resolution of 500 × 574.

To evaluate the segmentation performance of the method, we conducted experiments on two polyp segmentation datasets, Kvasir-SEG and CVC-ClinicDB. For each dataset, we randomly divided it into two subsets: 90 % for the training set and the remaining 10 % for the test set. To verify the generalizability of our model to data, we followed the experimental method of PraNet (Fan et al., 2020), extracting 900 and 550 images from the CVC-ClinicDB and Kvasir-SEG datasets, respectively, to form a training set of 1,450 images. Meanwhile, we used the CVC-ColonDB, CVC-300, and ETIS datasets as test sets to validate the model’s generalizability on different datasets. Table 1 summarizes the detailed information.

**TABLE 1:** The detailed information regarding the data divisions and dataset types.

Dataset	Year	Total	Train	Test	Type
Kvasir−SEG (Jha et al., 2020)	2020	1,000	900	100	Within dataset
CVC−ClinicDB (Bernal et al., 2015)	2015	612	550	62	Within dataset
CVC−ColonDB (Tajbakhsh et al., 2015)	2012	380	−	380	Cross dataset
CVC−300 (Vázquez et al., 2017)	2017	60	−	60	Cross dataset
ETIS (Silva et al., 2014)	2014	196	−	196	Cross dataset

#### 4.1.2 Training setup and experimental metrics

All of our experimental models are implemented under pytorch 2.0.0 and trained for 200 epochs on an RTX4090 graphics card with 24G of memory. Throughout the training regimen, we use four basic data augmentation techniques, random rotations, horizontal flips, vertical flips, and coarse masking, to enhance the model’s robustness to variations in the input data. And we use an Adam optimiser with the learning rate of 1e-4 and use the ReduceLROnPlateau learning rate scheduler. In our experiments, four separate experiments are conducted for each model, using four fixed random seeds: 42, 8, 36, and 120. The hyperparameters used in experiments are illustrated in Table 2. In the paper, all experimental data in the tables, unless otherwise specified, are the averages of these four experiments, with the variance calculated.

**TABLE 2:** Hyperparameters in experiments.

Epochs	Batchsize	Optimizer	LRschedule	Data augmentation	Loss function	Fixed random seeds
200	8	Adam	ReduceLROnPlateau	Random rotations, horizontal flips, vertical flips, coarse masking	Combine cross-entropy loss and dice loss	42, 8, 36, and 120

We combine cross-entropy loss and Dice loss as our assessment metrics for the loss function. To validate the effectiveness of our model, we have selected five metrics to evaluate the model’s performance from multiple perspectives: Dice Similarity Coefficient (Dice), Intersection over Union of polyp (IoUp), recall, Accuracy (ACC), and True Negative Ratio (TNR). Let FN, FP, TN, and TP denote false negatives, false positives, true negatives, and true positives, respectively. By definition, Dice, IoUp, recall, ACC, and TNR can be calculated by following Equations 25–29:

$$Dice=\frac{2TP}{FP+FN+2TP}$$ (25)

$$IoUp=\frac{TP}{FP+FN+TP}$$ (26)

$$recall=\frac{TP}{TP+FN}$$ (27)

$$ACC=\frac{TP+TN}{FP+TP+TN+FN}$$ (28)

$$TNR=\frac{TN}{FP+TN}$$ (29)

Generally, a superior segmentation method has larger values of Dice and IoUp.

### 4.2 Result

#### 4.2.1 Comparisons with state-of-the-art methods

To ensure an objective comparison, all the tested methods are selected from open-source works. Specifically, we select the following networks including Unet++ (Zhou et al., 2019), Unet3+ (Huang et al., 2020), Attention-UNet (Lian et al., 2018) (AttUNet), Context Encoder Network (Gu et al., 2019) (CENet), Local Global Interaction Network (Liu et al., 2023) (LGINet), Multi-scale Subtraction Network (Zhao et al., 2021) (MSNet), Duplex Contextual Relation Network (Yin et al., 2022) (DCRNet), Dual-Aggregation Transformer Network (Tang et al., 2023) (DuAT), Polyp-pvt (Dong et al., 2021), Transformer-based Residual Network (Jha et al., 2024) (TransNetR), Context axial reverse attention network (CaraNet) (Lou et al., 2022), as 11 state-of-the-art segmentation methods for comparison. To verify the validity of the correction, we performed a t-test between the state-of-the-art AFCNet and the three models that worked best in the other comparison experiments and calculated the p-value.

Specifically, the results in Table 3 show that our model achieved performance improvements of at least 1.72 % in Dice coefficient and 2.3 % in IoU on the ClinicDB dataset. To further validate the statistical significance of AFCNet, we conducted t-tests against the Top-3 baseline models (DCRNet, CaraNet, and DuAT). The results show that the p-values between AFCNet and the baselines were 0.0036, 0.0089, and 0.0059 for IoU, and 0.0179, 0.0182, and 0.005 for Dice, all of which are below the significance threshold (p < 0.05). The results demonstrate that the performance gains of AFCNet on the ClinicDB dataset are statistically significant.

**TABLE 3:** Comparison of our designed model AFCNet with currently popular methods on the CVC-ClinicDB dataset.([In %] and “ ± ” for variance).

Models	backbone	recall	TNR	Dice	ACC	IoUp
UNet++ (Zhou et al., 2019)	-	89.37±0.69	99.33±0.11	88.52±0.10	98.73±0.08	82.87±0.13
Unet3+ (Huang et al., 2020)	-	87.59±0.68	99.19±0.11	86.84±0.85	98.51±0.08	80.67±1.01
AttUNet (Lian et al., 2018)	-	89.49±1.15	99.22±0.13	88.38±1.37	98.58±0.01	82.53±1.27
CENet (Gu et al., 2019)	ResNet-34	93.46±0.60	99.35±0.08	91.76±0.70	99.07±0.08	86.67±1.05
LGINet (Liu et al., 2023)	-	88.65±1.50	99.03±0.21	87.65±1.08	98.58±0.11	81.52±1.62
DCRNet (Yin et al., 2022)	ResNet-34	94.13±1.66	99.52±0.13	92.83±0.75	99.24±0.08	88.37±0.60
MSNet (Zhao et al., 2021)	Res2Net-50	92.52±0.05	99.45±0.06	91.94±0.52	99.14±0.05	86.60±0.46
TransNetR (Jha et al., 2024)	ResNet-50	93.18±1.48	99.44±0.07	92.13±0.79	99.17±0.04	87.56±0.73
CaraNet (Lou et al., 2022)	Res2Net-50	95.21±0.84	99.47±0.06	93.08±0.65	99.22±0.04	88.37±0.64
Polyp−pvt (Yin et al., 2022)	PVT	95.48±0.73	99.29±0.14	92.15±0.99	99.13±0.10	87.03±1.24
DuAT (Tang et al., 2023)	PVT	94.93±0.81	99.49±0.11	93.06±0.48	99.26±0.05	88.29±0.71
AFCNet(ours)	Res2Net-50	94.54±0.96	99.61±0.07	94.48±0.22	99.33±0.07	89.88±0.33
AFCNet(ours)	ResNest-50	95.33±0.67	99.60±0.06	94.64±0.71	99.36±0.07	90.46±0.89
AFCNet(ours)	PVT	95.79±0.24	99.59±0.03	94.78±0.19	99.37±0.03	90.59±0.16

As shown in Table 4, AFCNet also demonstrated better performance on the Kvasir-SEG dataset, achieving improvements of 0.57 % in Dice and 0.94 % in IoU. We further performed t-tests against the Top-3 baselines (DuAT, Polyp-PVT, and MSNet), yielding p-values of 0.0027, 0.0143, and 0.0014 for IoU, and 0.017, 0.0382, and 0.001 for Dice, all significantly below 0.05. These statistical results confirm that AFCNet’s performance improvements on the Kvasir-SEG dataset are also statistically significant. In Table 5, we evaluate the inference time and model parameters of AFCNet.

**TABLE 4:** Comparison of our designed model AFCNet with currently popular methods on the Kvasir-SEG dataset.([In %] and “ ± ” for variance).

Models	backbone	recall	TNR	Dice	ACC	IoUp
UNet++ (Zhou et al., 2019)	-	86.12±0.79	98.32±0.13	85.57±1.09	96.00±0.27	78.60±1.40
Unet3+ (Huang et al., 2020)	-	82.94±0.55	97.72±0.42	81.02±1.35	94.81±0.34	72.77±1.60
AttUNet (Lian et al., 2018)	-	87.47±0.94	98.17±0.26	86.49±0.62	96.11±0.21	79.62±0.60
CENet (Gu et al., 2019)	ResNet-34	89.80±0.56	98.19±0.31	89.66±0.37	96.89±0.25	83.41±0.54
LGINet (Liu et al., 2023)	-	88.42±0.76	97.89±0.44	87.19±1.32	96.16±0.34	80.72±1.52
DCRNet (Yin et al., 2022)	ResNet-34	90.18±1.50	97.77±0.51	88.78±0.96	96.50±0.32	82.87±1.00
MSNet (Zhao et al., 2021)	Res2Net-50	89.91±0.95	98.58±0.39	89.41±0.72	96.79±0.22	84.01±0.75
TransNetR (Jha et al., 2024)	ResNet-50	89.25±0.83	98.30±0.33	88.57±0.38	96.52±0.15	82.35±0.44
CaraNet (Lou et al., 2022)	Res2Net-50	90.78±1.01	98.45±0.33	89.57±0.62	96.85±0.22	83.58±0.69
Polyp−pvt (Yin et al., 2022)	PVT	92.51±0.92	99.01±0.43	91.68±0.30	97.38±0.26	86.51±0.42
DuAT (Tang et al., 2023)	PVT	91.67±1.19	98.61±0.28	91.29±0.34	97.32±0.15	86.11±0.41
AFCNet(ours)	Res2Net-50	90.81±0.72	98.79±0.21	90.48±0.15	97.17±0.07	85.12±0.28
AFCNet(ours)	ResNest-50	92.10±0.84	98.76±0.22	91.44±0.30	97.49±0.07	86.13±0.38
AFCNet(ours)	PVT	92.51±0.59	98.74±0.18	92.35±0.49	97.55±0.10	87.53±0.30

**TABLE 5:** Computational efficiency comparison of AFCNet with different backbone networks. The table shows the computational complexity (GFLOPs), number of parameters, and inference speed (frames per second) for each configuration.

backbone	GFLOPs	Param	Inference(FPS)
AFCNet(Res2Net50)	8.043	29,939,345	50.81
AFCNet(ResNest50)	8.802	31,704,233	45.16
AFCNet(PVT)	9.243	28,393,217	33.82

**FIGURE 4:**
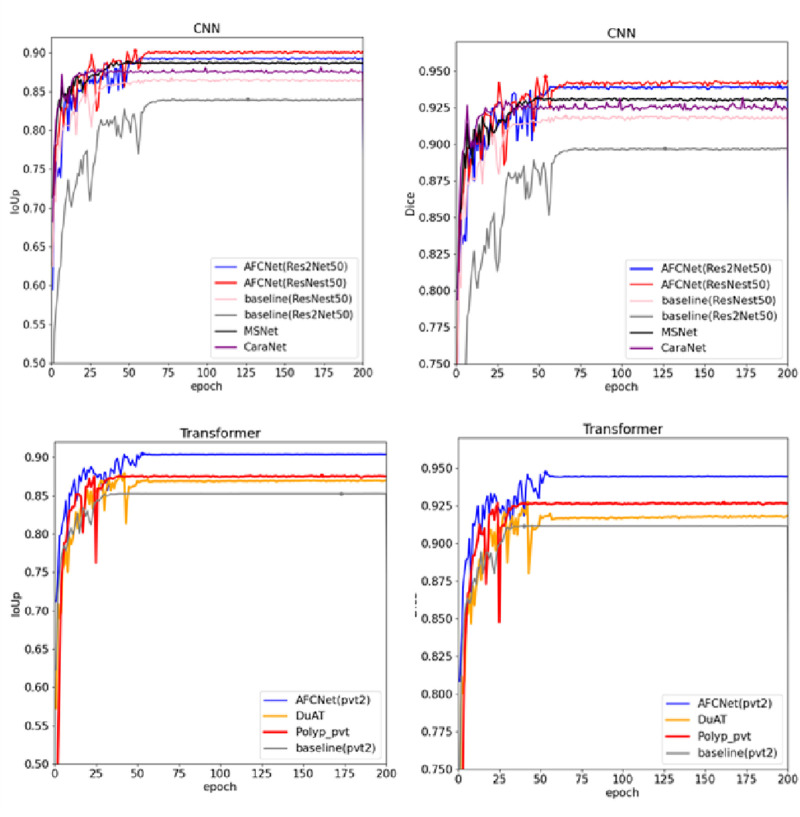
Change curves for the two KPIs when modeled using different backbones as encoders, as well as for the baseline model and two advanced models using the corresponding backbones on CVC-ClinicDB dataset.

**FIGURE 5:**
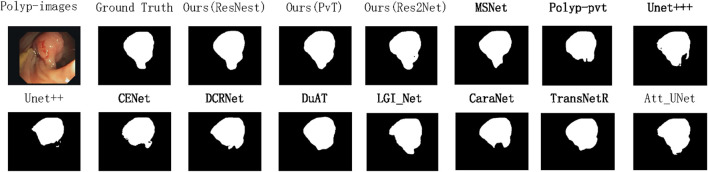
Qualitative results are used to compare the ground truth, our three methods, and eleven state-of-the-art methods on CVC-ClinicDB datasets.

**FIGURE 6:**
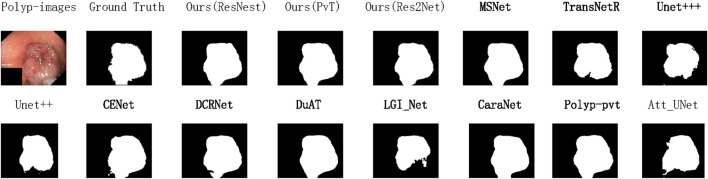
Qualitative results are used to compare the ground truth, our three methods, and eleven state-of-the-art methods on Kvasir-SEG datasets.

To demonstrate the state-of-the-art performance of our model, Figure 4 presents the variation curves of two key metrics (IoU and Dice) when using different backbone networks as the encoder. The results are categorized into two main groups: CNN-based backbones and Transformer-based backbones. For each category, we include performance curves of our model along with two state-of-the-art models using the same backbone technology and the baseline model for comparison. The curves clearly show that our model achieves optimal performance regardless of the backbone architecture. Based on previous experimental findings, our model demonstrates the best results when employing PVT as the backbone network. Therefore, for the data generalization experiments, we directly use the PVT-based configuration to compare with other models, as shown in Figures 5, 6. The polyps in the selected images exhibit characteristics such as irregular shapes, the presence of bubbles, and complex backgrounds.

To further evaluate the computational efficiency, we conducted comprehensive analyses on three backbone variants of AFCNet (Res2Net50, ResNest50, and PVT). As shown in Table 5, we systematically measured and compared several key metrics including parameter counts, computational complexity (GFLOPs), and inference speed (FPS) on GPU platforms. Additionally, we specifically analyzed the computational overhead of key components (MDCA, MAFF, and UFR modules) in Table 6. The experimental results demonstrate that while these modules introduce certain computational costs, they maintain an excellent balance between performance improvement and computational expense. These supplementary experiments not only validate AFCNet’s superiority in segmentation accuracy but also confirm its clinical applicability in terms of computational efficiency.

**TABLE 6:** Computational cost analysis of AFCNet with incremental module integration.

Models	GFLOPs	Param	Inference(FPS)
baseline	6.016	26,898,257	57.16
baseline+MAFF	7.049	28,364,177	51.37
baseline+MAFF+MDCA	7.43	28,808,849	51.63
baseline+MAFF+MDCA+UFR	8.043	29,939,345	50.81

#### 4.2.2 Generalisability experiments

The generalization ability of Computer-Aided Diagnosis (CAD) systems is crucial in clinical applications. To validate the generalization ability of AFCNet, we followed the experimental methodology of PraNet (Fan et al., 2020). We selected 550 images from CVC ClinicDB and 900 images from Kvasir, forming a training set of 1,450 images. To verify the network’s generalization performance, we used the entire ETIS, CVC ColonDB, and CVC-300 datasets as unseen data for testing. As shown in Table 7, Tables 8, 9, relative to the current popular networks, AFCNet improves Dice by 3.73 % , IoUp by 4.62 % on the ETIS dataset, and on the CVC-ColonDB dataset set, Dice improves by 0.91 % , IoUp improves by 0.71 % , and on the CVC-300 dataset, Dice improves by 0.46 % , IoUp improves by 0.94 % . It can be clearly seen that our method achieves the best results on all three datasets, which shows that our method has good learning ability with more robust generalization performance.

**TABLE 7:** Comparison of our designed model AFCNet with currently popular methods on the CVC-ColonDB dataset.([In %] and “ ± ” for variance).

Models	recall	TNR	Dice	ACC	IoUp
UNet++ (Zhou et al., 2019)	64.65±1.24	98.29±0.25	59.89±1.77	94.96±0.28	51.81±1.58
Unet3+ (Huang et al., 2020)	61.75±1.87	97.89±0.59	55.58±2.65	94.53±0.50	47.16±3.14
AttUNet (Lian et al., 2018)	64.86±1.04	98.69±0.25	61.36±0.96	95.35±0.13	53.51±1.14
CENet (Gu et al., 2019)	72.85±2.11	99.22±0.18	71.03±1.67	95.95±0.24	63.45±1.51
LGINet (Liu et al., 2023)	70.69±1.82	98.09±0.35	64.92±1.67	95.46±0.31	56.98±1.54
DCRNet (Yin et al., 2022)	77.48±4.07	98.60±0.66	73.67±1.99	96.13±0.17	65.68±1.62
MSNet (Zhao et al., 2021)	70.45±2.06	99.52±0.07	71.20±1.65	96.19±0.27	64.19±1.70
TransNetR (Jha et al., 2024)	64.79±1.76	99.59±0.07	66.23±1.53	95.69±0.04	59.29±1.41
CaraNet (Lou et al., 2022)	76.35±2.74	99.09±0.11	73.57±3.01	96.22±0.21	65.91±2.73
Polyp−pvt (Yin et al., 2022)	80.31±0.96	98.95±0.37	77.88±1.00	96.94±0.33	69.96±0.94
DuAT (Tang et al., 2023)	80.46±0.82	98.59±0.29	77.37±0.76	96.63±0.19	69.03±0.77
AFCNet	81.26±1.16	98.86±0.10	78.79±0.35	96.99±0.13	70.67±0.17

**TABLE 8:** Comparison of our designed model AFCNet with currently popular methods on the ETIS dataset.([In %] and “ ± ” for variance).

Models	recall	TNR	Dice	ACC	IoUp
UNet++ (Zhou et al., 2019)	43.42±3.88	98.72±0.16	39.31±4.23	96.88±0.19	33.70±3.58
Unet3+ (Huang et al., 2020)	59.05±3.14	98.24±1.13	56.44±1.93	97.18±1.02	48.99±1.87
AttUNet (Lian et al., 2018)	44.28±2.25	98.99±0.15	40.62±1.11	97.09±0.10	35.48±1.09
CENet (Gu et al., 2019)	66.98±4.20	98.62±0.59	62.32±2.61	97.85±0.46	55.29±1.91
LGINet (Liu et al., 2023)	47.90±2.32	98.18±0.64	42.76±3.91	96.70±0.72	37.27±3.65
DCRNet (Yin et al., 2022)	67.79±4.25	97.96±1.32	59.37±2.14	97.19±1.15	52.59±2.50
MSNet (Zhao et al., 2021)	73.35±3.56	99.10±3.86	69.08±2.06	98.50±0.32	61.92±1.54
TransNetR (Jha et al., 2024)	58.85±4.01	99.43±0.10	57.12±3.67	98.32±0.20	51.15±3.29
CaraNet (Lou et al., 2022)	83.01±3.86	97.19±1.28	68.86±2.04	96.84±1.16	60.52±1.93
Polyp−pvt (Yin et al., 2022)	82.10±1.62	98.47±0.44	73.00±1.97	98.21±0.41	64.64±2.22
DuAT (Tang et al., 2023)	78.97±1.19	98.69±0.19	72.43±2.04	98.40±0.19	62.95±2.75
AFCNet	83.09±2.56	98.81±0.26	76.73±0.91	98.51±0.17	69.26±0.56

**TABLE 9:** Comparison of our designed model AFCNet with currently popular methods on the CVC-300 dataset. ([In %] and “ ± ” for variance).

Models	recall	TNR	Dice	ACC	IoUp
UNet++ (Zhou et al., 2019)	80.64±3.17	98.95±0.32	73.01±1.53	98.27±0.28	64.20±1.52
Unet3+ (Huang et al., 2020)	79.44±3.63	98.11±1.04	68.24±3.32	97.51±0.87	58.95±3.60
AttUNet (Lian et al., 2018)	79.18±3.00	98.97±0.36	72.30±1.89	98.25±0.30	64.40±2.06
CENet (Gu et al., 2019)	90.14±4.11	99.15±0.36	84.60±0.80	98.89±0.31	77.07±0.99
LGINet (Liu et al., 2023)	88.32±2.78	98.74±0.34	78.86±1.64	98.48±0.24	70.09±1.37
DCRNet (Yin et al., 2022)	94.69±1.72	99.13±0.37	86.63±1.86	98.96±0.32	79.36±1.82
MSNet (Zhao et al., 2021)	93.08±0.81	99.49±0.12	88.78±0.73	99.28±0.10	81.57±1.01
TransNetR (Jha et al., 2024)	89.58±1.84	99.54±0.10	87.24±1.06	99.23±0.06	79.86±0.97
CaraNet (Lou et al., 2022)	96.16±0.54	99.09±0.19	86.74±0.41	99.00±0.16	79.14±0.47
Polyp−pvt (Yin et al., 2022)	94.37±0.36	99.36±0.11	87.63±0.58	99.19±0.10	80.27±0.81
DuAT (Tang et al., 2023)	94.21±1.16	99.01±0.32	86.44±0.61	98.85±0.28	79.19±0.43
AFCNet	94.54±0.46	99.55±0.08	89.24±0.53	99.38±0.04	82.51±0.55

#### 4.2.3 Ablation experiments

To systematically validate the effectiveness of each module, we designed a dual ablation study scheme:

We systematically integrated all proposed modules into three backbone networks (Res2Net50, ResNest50, and PvT2) to validate the architecture’s overall compatibility. All experiments were performed on the CVC-ClinicDB and Kvasir-SEG datasets. While preserving the complete hierarchical structure of the feature extraction backbone, we initially removed all modules to maintain only the basic U-shaped encoder-decoder framework, then sequentially incorporated the MAFF module, MDCA module, and UFR module. To specifically verify the effectiveness of the MAFF module’s structure, we conducted simplified ablation studies on the Res2Net50 backbone network followed by comprehensive experimental analysis. The results illustrated in Tables 10–13 are all obtained when Res2Net50 is backbone network.

**TABLE 10:** Ablation study of MAFF module variants on the ClinicDB dataset. ([In %] and “ ± ” for variance).

Models	recall	TNR	Dice	ACC	IoUp
baseline	90.40±0.52	99.21±0.15	89.44±0.88	98.86±0.11	83.57±1.28
MAFF(NoSubtraction)	91.66±3.63	99.45±1.04	90.99±0.77	98.95±0.10	85.88±0.95
MAFF(NoAddition)	79.44±3.63	98.11±1.04	68.24±3.32	97.51±0.87	88.25±0.61
MAFF	94.30±0.52	99.57±0.05	94.01±0.52	99.32±0.04	89.55±0.40

**TABLE 11:** Ablation study of MAFF module variants on the Kvasir-SEG dataset. ([In %] and “ ± ” for variance).

Models	recall	TNR	Dice	ACC	IoUp
baseline	88.20±0.67	98.44±0.09	87.94±0.65	96.51±0.25	81.37±1.08
MAFF(NoSubtraction)	90.18±1.50	97.77±0.51	88.78±0.96	96.50±0.32	82.87±1.00
MAFF(NoAddition)	89.85±0.86	98.60±0.29	89.44±0.29	97.06±0.29	83.91±0.53
MAFF	89.63±0.77	98.78±0.32	89.78±0.24	97.01±0.17	84.20±0.15

**TABLE 12:** Performance comparison of segmentation using MDCA, CPCA, and CoordAttention on CVC-CLinicDB dataset. ([In %] and “ ± ” for variance).

Models	recall	TNR	Dice	ACC	IoUp
ChannelPriorConvolutionalAttention	92.17±2.02	99.44±0.004	91.65±2.28	99.10±0.005	86.63±1.76
CoordAttention	91.41±7.03	99.51±0.006	90.40±2.64	99.06±0.02	85.34±2.60
MDCA	94.54±0.96	99.61±0.07	94.48±0.22	99.34±0.06	89.88±0.33

**TABLE 13:** Performance comparison of segmentation using MDCA, CPCA, and CoordAttention on the CVC-CLinicDB dataset. ([In %] and “ ± ” for variance).

Models	recall	TNR	Dice	ACC	IoUp
ChannelPriorConvolutionalAttention	89.97±1.85	98.71±0.007	89.87±0.73	97.09±0.06	84.35±1.05
CoordAttention	89.98±0.19	98.65±0.07	90.28±0.19	97.22±0.02	84.68±0.23
MDCA	90.81±0.72	98.74±0.18	90.48±0.15	97.17±0.07	85.12±0.28

##### 4.2.3.1 Effectiveness of MAFF module

In order to verify the effectiveness of the MAFF module in the model, we input the multilayer features extracted from the backbone network directly into the MAFF module and then up-sampled them directly. As can be seen from Table 14, all the metrics of the model with the addition of the MAFF module are significantly better than the baseline model, both on different datasets and different backbone network architectures. This is because the MAFF module is able to dynamically balance the impact of the two feature fusion methods on the final feature representation through the trainable parameters, thus making the two methods complementary to each other.

**TABLE 14:** Ablation study for the various modules with different backbone on the Kvasir-SEG dataset. ([In %] and “ ± ” for variance).

Backbone	Models	recall	TNR	Dice	ACC	IoUp
	baseline	90.40±0.52	99.21±0.15	89.44±0.88	98.86±0.11	83.57±1.28
Res2Net-50	+MAFF	94.30±0.52	99.57±0.05	94.01±0.52	99.32±0.04	89.55±0.40
	+MAFF+MDCA	94.33±0.68	99.63±0.06	94.33±0.10	99.33±0.07	89.70±0.29
	+MAFF+MDCA+UFR	94.54±0.96	99.61±0.07	94.48±0.22	99.34±0.06	89.88±0.33
	baseline	92.15±0.64	99.40±0.04	91.18±0.50	99.04±0.04	85.8±0.56
ResNest-50	+MAFF	95.13±0.71	99.58±0.07	94.34±0.72	99.34±0.05	90.07±0.83
	+MAFF+MDCA	95.16±0.75	99.60±0.06	94.64±0.37	99.36±0.07	90.36±0.63
	+MAFF+MDCA+UFR	95.33±0.67	99.61±0.05	94.74±0.71	99.37±0.06	90.47±0.89
	baseline	91.10±0.64	99.40±0.04	91.48±0.82	99.04±0.04	85.94±0.82
PVT	+MAFF	95.85±0.42	99.55±0.05	94.11±0.35	99.33±0.02	89.95±0.34
	+MAFF+MDCA	95.60±0.61	99.59±0.05	94.53±0.35	99.36±0.02	90.31±0.33
	+MAFF+MDCA+UFR	95.79±0.24	99.59±0.03	94.78±0.19	99.37±0.03	90.59±0.16

The MAFF module is validated as an effective multi-scale feature fusion method. In addition to this basic ablation experiment, in order to explore the structural validity of the MAFF module, we conducted systematic ablation experiments comparing three configurations: (1) the baseline model without MAFF, (2) MAFF with only additive units, and (3) MAFF with only subtractive units. The experimental results from Tables 10, 11 show that the full MAFF module significantly outperforms the variant model in all evaluation metrics (ClinicDB dataset: 4.57 % improvement in Dice and 5.98 % improvement in IoU; Kvasir-SEG dataset: 1.84 % improvement in Dice and 2.83 % improvement in IoU) and performs consistently across different datasets and backbone networks. According to work (Song et al., 2022), MSNet uses Subtractive Units (SU) in the Decoder part to generate difference features between adjacent levels of the network, which can easily lead to the loss of edge information for smaller polyps and affect segmentation accuracy. According to the work (Zhou et al., 2018), addition preserves semantic consistency without losing information.

##### 4.2.3.2 Effectiveness of the MDCA module

After the model is added to the MDCA module, as shown in Tables 14, 15, the segmentation ability of the model has a more obvious improvement, which indicates that the important information in the image can be well extracted by our MDCA module, this is because the convolution with different orientations and sizes can capture a wider range of feature information, and is more sensitive to the targets with complex shapes, and can also be used with the MAFF module’s fusion mechanism, thus enhancing the model’s ability to represent image details and context.

**TABLE 15:** Ablation study for the various modules with different backbone on Kvasir-SEG dataset. ([In %] and “ ± ” for variance).

Backbone	Models	recall	TNR	Dice	ACC	IoUp
	baseline	88.20±0.67	98.44±0.09	87.94±0.65	96.51±0.25	81.37±1.08
Res2Net-50	+MAFF	89.63±0.77	98.78±0.32	89.78±0.24	97.01±0.17	84.20±0.15
	+MAFF+MDCA	90.25±0.42	98.71±0.22	90.15±0.47	97.13±0.20	84.88±0.54
	+MAFF+MDCA+UFR	90.81±0.72	98.74±0.18	90.48±0.15	97.17±0.07	85.12±0.28
	baseline	89.02±0.82	98.51±0.11	88.72±0.58	96.76±0.17	82.26±0.46
ResNest-50	+MAFF	91.30±1.01	98.74±0.11	90.82±0.46	97.31±0.10	85.34±0.44
	+MAFF+MDCA	92.10±0.84	98.69±0.12	91.35±0.42	97.49±0.07	85.97±0.57
	+MAFF+MDCA+UFR	92.34±0.61	98.76±0.22	91.44±0.30	97.49±0.07	86.13±0.38
	baseline	91.10±0.07	98.57±0.40	90.24±0.66	97.10±0.29	84.25±0.93
PVT	+MAFF	91.79±0.15	98.79±0.19	91.93±0.50	97.41±0.20	86.92±0.57
	+MAFF+MDCA	91.87±0.15	98.89±0.32	92.15±0.30	97.55±0.19	87.25±0.24
	+MAFF+MDCA+UFR	92.51±0.59	98.74±0.18	92.35±0.49	97.55±0.10	87.53±0.30

To validate the effectiveness of the MDCA module in multi-scale feature extraction, we designed a comparative experiment. In this experiment, while keeping the network structure unchanged, the MDCA module was replaced with the CPCA and CoordAttention modules for performance comparison. As shown in Tables 12, 13, the experimental results demonstrate that MDCA outperforms the competing methods in polyp boundary segmentation accuracy. This highlights the superiority of our design for complex medical image segmentation tasks.

##### 4.2.3.3 Effectiveness of the UFR module

The UFR module filters the information in the up-sampling stage through the gating mechanism, and in terms of the model effect, Tables 14, 15 demonstrates that the UFR can filter and fuse the fused features very well, so as to optimize the segmentation capability of the model in a stable manner.

### 4.3 Discussion

The proposed architecture in this paper is an end-to-end processing framework, meaning that image analysis is completed within a single framework (Biju et al., 2024). An alternative approach employs a step-by-step construction of deep learning models, such as preprocessing the image before performing the analysis (Qian et al., 2020; Vijayalakshmi and Sasithradevi, 2024). Both methods have their advantages. End-to-end deep learning models reduce the complexity of intermediate steps and make more efficient use of computational and memory resources. Step-by-step deep learning models, on the other hand, offer better interpretability, task flexibility, and advantages in modular expansion. Future research could focus on further integrating the strengths of both paradigms to develop hybrid systems that are flexible and robust.

This work was trained and tested on an RTX 4090 GPU, a type of hardware that is still not feasible to deploy on many resource-constrained embedded platforms. Therefore, another important issue for future research is how to effectively improve the execution efficiency of polyp segmentation methods, in order to further reduce their operational costs and enhance real-time performance. Compression techniques, such as quantization and pruning (Frantar et al., 2022), along with the use of lightweight architectures (Ahamed et al., 2023b; Ahamed et al., 2025), can help reduce model size by exploiting the sparsity of effective model parameters. However, relying on a single model attribute for performance optimization has its limitations. A more comprehensive approach that integrates multiple optimization strategies is likely to yield better results. For example, in PowerInfer (Song et al., 2024), the authors successfully combined the model’s sparsity with the challenge of efficiently deploying the model across heterogeneous resources, achieving significant performance improvements. Our future work will also focus on exploring hybrid techniques for model optimization.

## 5 Conclusion

This paper proposes a novel polyp segmentation network, AFCNet. It is based on convolutional attention and adaptive multi-scale feature fusion. In the feature extraction and enhancement stage, the MDCA module captures broader contextual information from images. At the same time, it increases the weights of important features. By simplifying the deepest layer features in the backbone network, a more efficient architecture is achieved. During the feature fusion stage, the MAFF module integrates features from different layers. It dynamically balances multiple fusion strategies. This process continuously improves the model’s ability to capture both global and detailed information. Therefore, superior multi-scale feature fusion performance is achieved. In the upsampling stage, the UFR module filters and guides the final fused features. In the experimental section, we compare our method with 11 state-of-the-art polyp segmentation approaches. We also evaluate the module’s generalizability by integrating it with different backbone networks. The results demonstrate that our method achieves the best performance. It also maintains excellent generalization and adaptability.

## Data Availability

The original contributions presented in the study are included in the article/supplementary material, further inquiries can be directed to the corresponding author.
